# Mechanisms of Concentric Ring Electrodes in Tuning the Performance of Z-Cut Lithium Niobate Ultrasonic Transducers

**DOI:** 10.3390/s26020481

**Published:** 2026-01-11

**Authors:** Xuesheng Ouyang, Liang Zhong, Jun Zhou, Guanghua Li, Hui Hu, Kai Wang, Yizhe Jia, Hao Dai, Jinlong Mo, Kaiyan Huang, Jishuo Wang

**Affiliations:** 1 Key Laboratory of Testing Technology for Manufacturing Process MOE, Southwest University of Science and Technology, Mianyang 621010, China; xueshengouyang@mails.swust.edu.cn (X.O.); zhongliang@swust.edu.cn (L.Z.); fayhu@swust.edu.cn (H.H.); yizhejia@mails.swust.edu.cn (Y.J.); jinlong_mo@mails.swust.edu.cn (J.M.); huangkaiyan@swust.edu.cn (K.H.); jswang@swust.edu.cn (J.W.); 2 Department of Mechatronic Engineering, Sichuan Electronic Mechanic Vocational College, Mianyang 621023, China; 3 School of Mechanical Engineering, Hebei University of Technology, Tianjin 300401, China; 4 Henan Key Laboratory of Underwater Intelligent Equipment, The 713th Research Institute of China State Shipbuilding Corporation Limited, Zhengzhou 450015, China; lghuacsic@163.com (G.L.); wangkai24@buaa.edu.cn (K.W.); 5 School of Information and Control Engineering, Southwest University of Science and Technology, Mianyang 621000, China; houal@mails.swust.edu.cn

**Keywords:** Z-cut lithium niobate, concentric ring electrodes, ultrasonic transducer

## Abstract

Z-cut lithium niobate single crystal demonstrates considerable promise for contact-based ultrasonic nondestructive testing and structural health monitoring (SHM) transducers due to its high piezoelectric coefficients, strong electromechanical coupling capability, and environmentally friendly lead-free composition. As a simulation-based theoretical exploration, this study systematically investigates the impact of gap spacing and electrode width in concentric ring configurations on the resonant characteristics and pulse-echo response of ultrasonic transducers by establishing a parametrized finite element model. Numerical simulations reveal that electrode geometry plays a critical role in determining both the effective electromechanical coupling coefficient and echo signal strength. Optimizing the electrode ring width achieved an effective electromechanical coupling coefficient ($k_{\mathrm{eff}}$) of 35.2%, while systematic enlargement of the electrode gap further enhanced this value to 50.8%. The study also demonstrates that optimized ring width and adjusted electrode spacing increased the echo signal’s peak-to-peak amplitude ($V_{pp}$) by factors of 4.94 and 2.03, respectively, compared to the poorest-performing configuration within each parameter group. This study establishes that precise design of concentric electrode configurations serves as an effective strategy for tuning lithium niobate ultrasonic transducer characteristics, providing critical design guidelines for developing high-performance ultrasonic transducers for solid medium coupling.

## 1. Introduction

Ultrasonic nondestructive testing technology serves as a cornerstone of modern industrial quality assurance systems [1,2], playing an indispensable role in high-reliability sectors including aerospace, nuclear facilities, and rail transportation [3,4,5,6,7]. This technique enables precise characterization of component flaws and material properties through interactions between high-frequency acoustic waves and material internals, with performance enhancement being fundamentally contingent upon innovations in piezoelectric transducer materials and continuous optimization of structural design [8,9]. While lead zirconate titanate (PZT) ceramics exhibit outstanding piezoelectric properties [10,11], their practical implementation faces several technical constraints. For instance, PZT-based transducers typically demonstrate large dimensions, high driving power consumption, and poor compatibility with modern microelectronic processes, thereby constraining their application potential in compact devices [12,13]. More critically, the inherently high dielectric constant of PZT materials introduces significant parasitic capacitance effects in circuits, consequently degrading electromechanical conversion efficiency and compromising signal integrity [14]. Furthermore, the inherent environmental and health hazards associated with lead content in PZT throughout its lifecycle have generated increasing concern [15,16]. Consequently, developing novel piezoelectric materials that integrate superior comprehensive performance, excellent integration compatibility, and environmental friendliness has emerged as a critical pathway for advancing ultrasonic technologies [17,18,19].

Lithium niobate single crystals have emerged as a highly promising candidate due to their distinctive combination of advantageous properties [20,21]. In contrast to conventional PZT materials, lithium niobate not only possesses completely lead-free environmental advantages but also exhibits pronounced anisotropy, high piezoelectric coefficients, exceptional thermal stability, and remarkable electromechanical coupling capabilities [22,23,24,25]. Leveraging its superior piezoelectric and acoustic properties, lithium niobate crystals have enabled the establishment of mature technology platforms for both surface acoustic wave and bulk acoustic wave devices, with well-developed design methodologies and application systems [26,27,28,29].

Recent advances in lithium niobate devices through optimized crystal cuts and innovative interdigital electrode designs have demonstrated significant improvements in key performance metrics including filter insertion loss, out-of-band rejection, and power handling capacity [30,31,32,33]. These achievements underscore that both crystal orientation and electrode configuration serve as pivotal design degrees of freedom for performance engineering. By governing vibration modes and energy distribution, they fundamentally determine the ultimate device characteristics. For instance, Lu et al. [34] demonstrated a seminal work employing thin-film lithium niobate with lateral field excitation to fabricate a piezoelectric micromachined ultrasonic transducer, achieving a 4.2% electromechanical coupling coefficient with notably high quality factor at 7.6 MHz. However, the strong dependence of their lateral field excitation architecture on specific crystal orientations and electrode patterning inherently constrains design flexibility. Xie et al. [35] devised a Z-cut lithium niobate thin-film lateral-field-excited bulk acoustic resonator employing pentagonal spiral electrodes, which attained 21.3% effective electromechanical coupling coefficient at 5.43 GHz while substantially suppressing spurious modes. However, this electrode configuration incurred increased resistive losses that degraded the device’s quality factor. Liu et al. [36] effectively tailored resonant modes and electromechanical coupling characteristics in X-cut LiNbO_3_ thin-film resonators through strategic electrode rotation, significantly enhancing filter design flexibility. Nevertheless, this approach continues to face persistent challenges in spurious mode suppression and environmental stability assurance. Wen et al. [37] developed a lateral-field-excited bulk acoustic resonator incorporating scattering vias within the electrode structure, achieving substantial suppression of spurious modes with 21.80% electromechanical coupling coefficient. While significant progress has been achieved in communication devices, research focus has primarily centered on optimizing high-frequency resonance and filtering performance. However, for mid-to-low frequency applications such as contact-based nondestructive testing and structural health monitoring, systematic investigation into the fundamental correlations between geometric parameters of concentric ring electrodes, vibrational modes, and acoustic field behavior in Z-cut lithium niobate remains limited. Furthermore, compared to conventional thickness-oriented electrode configurations, this lateral field excitation architecture monolithically integrates the excitation components on a single crystal surface, eliminating the need for complex alignment processes required for through-thickness electrodes. This integration significantly simplifies device structure and reduces manufacturing costs. While prior studies have demonstrated the applicability of lateral field excitation in various devices, the research focus has predominantly centered on high-frequency communication devices [35,36,37,38,39]. A systematic understanding of how electrode geometry governs acoustic output performance in MHz-range ultrasonic transducers remains underdeveloped.

Accordingly, this study employs Z-cut lithium niobate to systematically investigate the control mechanisms of gap spacing and ring width in concentric electrode configurations on the performance of MHz-range ultrasonic transducers. Through the development of a parametric finite element model, we comprehensively analyze how electrode geometry influences lateral electric field distribution, modal coupling characteristics, and acoustic output. The results demonstrate that optimizing the electrode configuration can effectively enhance the electromechanical coupling efficiency and echo signal strength of the device, providing new theoretical foundations and structural optimization strategies for designing high-performance lithium niobate ultrasonic transducers for contact-based nondestructive testing and structural health monitoring.

## 2. Theoretical Analysis

For Z-cut lithium niobate wafers, the crystallographic z-axis is oriented normal to the wafer surface. Conventional thickness-field excitation employs electrode configurations on opposing surfaces where the electric field aligns parallel to the thickness direction, primarily exciting pure thickness-extensional vibration modes [40]. In contrast, the concentric ring electrode structure adopted in this work is patterned on a single crystal surface, generating electric fields predominantly confined to the lateral plane, representing a typical lateral-field excitation scheme.

Under lateral-field excitation, the applied electric field orientation differs from the crystal’s polarization direction, resulting in the simultaneous activation of multiple piezoelectric coefficients within the constitutive relations. In this case, the piezoelectric constitutive equations should be expressed in the following form [41,42]:

(1) $$\left\{\begin{array}{c} T_{p}=c_{pq}^{E}S_{q}-e_{kp}E_{k}\\ D_{i}=e_{iq}S_{q}+\varepsilon_{ik}^{S}E_{k} \end{array}\right.$$

where $T_{p}$ are the stress tensor components; $c_{pq}^{E}$ is the elastic stiffness matrix under constant electric field; $S_{q}$ are the strain tensor components; $e_{kp}$ is the piezoelectric stress coefficient matrix; $E_{k}$ are the electric field components; $D_{i}$ are the electric displacement components; $\varepsilon_{ik}^{S}$ is the permittivity matrix under constant strain.

For Z-cut lithium niobate crystals, the non-zero piezoelectric stress constants relevant to lateral field excitation primarily include $e_{15}$, $e_{22}$, $e_{31}$, and $e_{33}$. By expanding the constitutive equations and specifically analyzing the stress components driven by lateral electric fields $E_{x}$ and $E_{y}$, we can elucidate the excitation mechanisms of different vibration modes.

The lateral electric field component $E_{x}$ directly couples through the piezoelectric coefficient $e_{15}$ to generate shear stress $T_{5}$, thereby exciting the thickness-shear vibration mode:

(2) $$T_{5}=c_{55}^{E}S_{5}-e_{15}E_{x}$$

Simultaneously, the lateral electric field components $E_{x}$ and $E_{y}$ couple through piezoelectric coefficients $e_{31}$ and $e_{33}$ to generate in-plane stresses $T_{1}$ and $T_{2}$, consequently exciting in-plane vibration modes such as radial extension:

(3) $$\left\{\begin{array}{c} T_{1}=c_{11}^{E}S_{1}+c_{12}^{E}S_{2}-e_{31}E_{x}\\ T_{2}=c_{12}^{E}S_{1}+c_{11}^{E}S_{2}-e_{33}E_{y} \end{array}\right.$$

The fundamental resonance frequency $f_{TS}$ of the thickness-shear mode is governed by the wafer thickness t and the material’s shear wave velocity $v_{s}$, with their theoretical relationship satisfying:

(4) $$f_{TS}=\frac{v_{s}}{2t}$$

The effective electromechanical coupling coefficient ($k_{\mathrm{eff}}$) is a key parameter for evaluating the electromechanical energy conversion efficiency of transducers, whose value is determined by the resonance frequency ($f_{r}$) and anti-resonance frequency ($f_{a}$) through the following relation:

(5) $$k_{\mathrm{eff}}=\sqrt{\frac{\pi }{2}\cdot \frac{f_{r}}{f_{a}}\cdot tan(\frac{\pi }{2}\cdot \frac{f_{a}-f_{r}}{f_{a}})}$$

The magnitude of this parameter directly reflects the coupling strength between electrical energy and mechanical vibration energy. In lateral-field-excited concentric ring electrode structures, the geometric parameters of the electrodes govern the spatial distribution of the lateral electric field, thereby influencing vibration mode excitation efficiency and intermodal coupling strength, ultimately determining the $k_{\mathrm{eff}}$ value and the overall transducer performance.

## 3. Modeling and Simulation

### 3.1. Modeling of Z-Cut Lithium Niobate Piezoelectric Wafer

**Figure 1:**
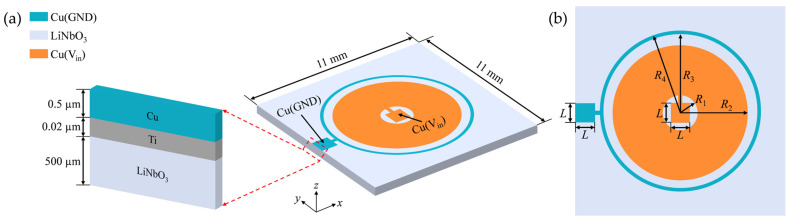
(**a**) Schematic of the piezoelectric wafer stack structure; (**b**) Schematic of the electrode configuration.

To accurately capture the full anisotropic characteristics and realistic electromechanical field distribution of the Z-cut lithium niobate crystal, this section establishes a 3D parametric finite element model of the piezoelectric wafer to systematically investigate how the geometric configuration of concentric ring electrodes affects its resonant characteristics and electroacoustic response. The core layered structure of the model is shown in Figure 1a.

The model utilizes a square Z-cut lithium niobate wafer with 11 mm side length and 500 μm thickness as the piezoelectric layer. A pair of concentric ring electrodes is patterned on the top surface, with the geometric configuration illustrated in Figure 1b, where L denotes the side length of the square bonding pad, $R_{1}$ and $R_{2}$ represent the inner and outer radii of the positive electrode, while $R_{3}$ and $R_{4}$ correspond to the inner and outer radii of the negative electrode, respectively. The electrodes are constructed from 0.5 μm thick copper. To address potential inadequate adhesion associated with direct copper-lithium niobate bonding, a 0.02 μm thick titanium interlayer is incorporated between the electrodes and the piezoelectric layer in the model. This adhesion layer is confined to the region directly beneath the electrodes, thereby enhancing the simulation fidelity of interfacial bonding and enabling more accurate prediction of practical device performance.

To elucidate the individual effects of electrode geometric parameters on vibration modes, we designed and conducted two distinct sets of parametric simulation studies.

The first set of experiments aims to investigate the influence of electrode ring width on device performance. The experimental design maintains a constant edge-to-edge gap between the positive (V_in_, excitation terminal) and negative (GND, ground terminal) electrodes (i.e., $R_{3\;}\;{-\;R}_{2\;}$= 0.5 mm). Under this constraint, with the inner ring’s inner radius $R_{1}$ and the outer ring’s outer radius $R_{4\;}$ fixed, the values of $R_{2}$ and $R_{3}$ were synchronously reduced in 0.5 mm steps (while satisfying the constraint $R_{2\;}>R_{1\;}$). This process generated a series of electrode configurations characterized by progressively decreasing ring widths but a constant inter-electrode gap. The specific parameters are listed in Table S1 (Supplementary Materials).

The second set of experiments was designed to investigate the effect of electrode spacing on device performance. This series systematically varies the inter-electrode distance by maintaining fixed negative electrode dimensions while adjusting the positive electrode position. Specifically, with the inner ring’s inner radius $R_{1\;}$ held constant, the inner ring’s outer radius $R_{2}$ was progressively reduced in 0.5 mm increments (while consistently satisfying the geometric constraint $R_{2\;}-R_{1\;}$> 0.5 mm). This adjustment progressively increases the radial separation between the positive and negative electrodes. The corresponding parameter configurations are detailed in Table S2 of the Supplementary Materials. This parametric scanning approach enables effective identification of the dominant effects of different geometric factors on resonant characteristics.

The material constants of the Z-cut lithium niobate single crystal employed in this simulation study are listed below [43,44]. The compliance matrix is:

(6) $$\begin{array}{l} s=\left[\begin{array}{cccccc} s_{11} & s_{12} & s_{13} & s_{14} & 0 & 0\\ s_{12} & s_{11} & s_{13} & -s_{14} & 0 & 0\\ s_{13} & s_{13} & s_{33} & 0 & 0 & 0\\ s_{14} & -s_{14} & 0 & s_{44} & 0 & 0\\ 0 & 0 & 0 & 0 & s_{44} & 2s_{14}\\ 0 & 0 & 0 & 0 & 2s_{14} & 2(s_{11}-s_{12}) \end{array}\right]\\ =\left[\begin{array}{cccccc} 5.78 & -1.01 & -1.47 & -1.02 & 0 & 0\\ -1.01 & 5.78 & -1.47 & 1.02 & 0 & 0\\ -1.47 & -1.47 & 5.02 & 0 & 0 & 0\\ -1.02 & 1.02 & 0 & 17 & 0 & 0\\ 0 & 0 & 0 & 0 & 17 & -2.04\\ 0 & 0 & 0 & 0 & -2.04 & 13.6 \end{array}\right]10^{-12}[\frac{1}{Pa}]. \end{array}$$

The piezoelectric coupling matrix is:

(7) $$d=\left[\begin{array}{cccccc} 0 & 0 & 0 & 0 & d_{15} & -2d_{22}\\ -d_{22} & d_{22} & 0 & d_{15} & 0 & 0\\ d_{31} & d_{31} & d_{33} & 0 & 0 & 0 \end{array}\right]\;=\left[\begin{array}{cccccc} 0 & 0 & 0 & 0 & 68 & -42\\ -21 & 21 & 0 & 68 & 0 & 0\\ -1 & -1 & 6 & 0 & 0 & 0 \end{array}\right]10^{-12}[C/N].$$

The relative permittivity matrix is:

(8) $$\varepsilon =\left[\begin{array}{ccc} \varepsilon_{11} & 0 & 0\\ 0 & \varepsilon_{11} & 0\\ 0 & 0 & \varepsilon_{33} \end{array}\right]=\left[\begin{array}{ccc} 84 & 0 & 0\\ 0 & 84 & 0\\ 0 & 0 & 30 \end{array}\right].$$

Regarding the simulation setup, frequency-domain analysis was performed first. The negative electrode was defined as a ground boundary, while a terminal excitation with an amplitude of 1 V was applied to the positive electrode. A frequency sweep from 0.02 MHz to 10 MHz was implemented with a step size of 0.005 MHz to accurately capture the impedance characteristics and resonant spectrum of the system.

### 3.2. Modeling of the Z-Cut Lithium Niobate Ultrasonic Transducer

Given the coexistence of sub-micron electrodes and millimeter-scale acoustic structures in MHz-range transducers, practical fabrication requires a “Hybrid Micro-Macroscopic Fabrication Process.” The specific workflow proceeds as follows: First, in the micro-fabrication stage, utilizing a precision-polished Z-cut lithium niobate wafer as the substrate, standard ultraviolet (UV) lithography and magnetron sputtering processes are employed to define high-precision concentric ring electrode structures with sub-micron thickness on the wafer surface. Subsequently, entering the macro-integration stage, following wire bonding, a precision casting and lapping process is applied to the radiation surface to fabricate an alumina/epoxy matching layer with a specific millimeter-scale thickness. Finally, a millimeter-thick tungsten/epoxy backing layer is formed on the rear side of the device via mold casting and curing [45,46,47].

To evaluate the practical performance of the ultrasonic transducer designed based on the aforementioned fabrication framework, a corresponding two-dimensional finite element model of the ultrasonic transducer was established in this section. Based on the three-dimensional eigenmode analysis of the piezoelectric element (the detailed results of which will be discussed in Section 4.1), the displacement of Z-cut lithium niobate at resonance is predominantly governed by the shear component parallel to the crystallographic Y-axis. Given this physical phenomenon, to balance computational efficiency with a focus on wave propagation characteristics along the principal energy axis, this model constructs the 2D geometry by selecting the cross-section along the crystal Y-axis (i.e., the YZ plane). Serving as an effective projection of the 3D physical field onto the dominant vibration cross-section, this 2D model accurately captures the core electromechanical coupling and wave propagation features, thereby effectively revealing the modulation laws of electrode geometric parameters on echo energy intensity and acoustic response.

**Figure 2:**
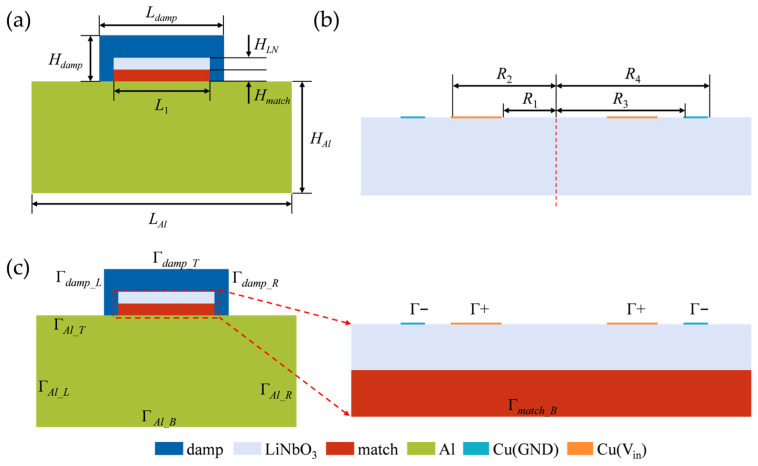
Two-dimensional model of the ultrasonic transducer. (**a**) Schematic of the geometric parameters; (**b**) Electrode configuration. (**c**) Schematic diagram of the boundary condition layout.

The two-dimensional geometry of the transducer is shown in Figure 2a. The simulation model incorporates the following key geometric parameters: aluminum block length $L_{Al}$ and height $H_{Al}$, matching layer height $H_{match}$, lithium niobate layer height $H_{LN}$, backing layer length $L_{damp}$ and height $H_{damp}$, and the shared length $L_{1}$ of the lithium niobate and matching layer. The specific dimensional parameters are summarized in Table S3 (Supplementary Materials). For electrode modeling within the two-dimensional framework, the three-dimensional concentric ring electrode structure is simplified as a series of line segments assigned to specific boundaries on the top surface of the lithium niobate layer, as depicted in Figure 2b, where V_in_ denotes the excitation terminal and GND represents the ground terminal. The radial positions and dimensions of these line-segment electrodes strictly adhere to the parameters defined in Tables S1 and S2, thereby representing the electrode geometry and the resulting lateral electric field distribution within the two-dimensional representation.

For material modeling, the matching layer and backing layer were modeled using Al_2_O_3_/epoxy and tungsten/epoxy composite materials, respectively. By varying the volume fraction of the filler materials, the acoustic impedance of the composites can be continuously adjusted, thereby optimizing the acoustic energy transmission efficiency.

The equivalent elastic parameters of these two-phase composite materials were derived based on the Devaney theoretical model [48] to ensure physical rationality of the material response. The detailed material property parameters for each simulation layer are summarized in Table S4 (Supplementary Materials).

A modulated Gaussian pulse signal with a center frequency of $f_{0}$ was applied to the positive electrode as the excitation source. Its expression is given by:

(9) $$V(t)=10\cdot e^{-{\left(\frac{t-2T_{0}}{T_{0}/2}\right)}^{2}}\cdot sin(2\pi f_{0}t)$$

where $T_{0}$ represents the characteristic time constant defined as $T_{0}=1/f_{0}$. According to Equation (4) and the material parameters provided in Table S4, the theoretical fundamental resonance frequency of the thickness-shear mode for a 500 μm thick Z-cut lithium niobate wafer is approximately 3.98 MHz. However, in the actual device structure, the loading effect from the top-surface electrodes and the titanium adhesion layer introduces additional mass loading and stiffness, consequently resulting in a primary resonant frequency of the device that is slightly lower than the ideal theoretical value. To ensure the excitation signal effectively covers and stimulates the actual operational modes of the transducer, the center frequency $f_{0}$ of the excitation signal was set to 3.75 MHz in this study.

In the model, boundaries Γ+ and Γ− are used to apply the excitation signal and define the ground condition, respectively. The overall layout of the boundary conditions is presented in Figure 2c. It should be noted that the simulation model represents an SHM application scenario where the transducer is rigidly bonded to the surface of the test block. Therefore, an acoustic continuity boundary condition is applied at the contact interface (Γ*_match_B_*) between the transducer and the aluminum block to simulate the effective transmission of shear waves between solid media, and the specific configuration parameters are listed in Table 1.

**Figure 3:**
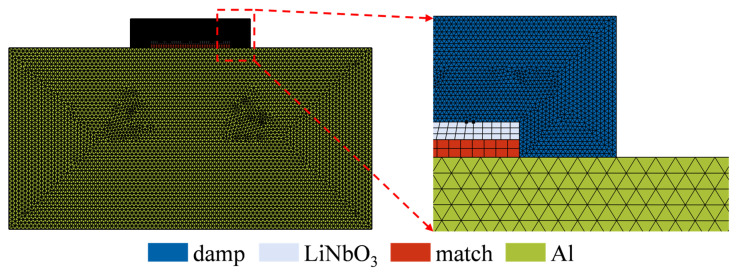
Model mesh discretization.

In numerical simulations of wave propagation problems, the rationality of mesh generation directly determines computational accuracy, with the core requirement being the effective resolution of acoustic wavelengths. Since wavelength is jointly determined by material sound velocity and excitation frequency, regions with lower sound velocities require finer mesh densities to accurately capture wavefield evolution details. This study employs the discontinuous Galerkin Finite Element Method (dG-FEM) for numerical solution, which typically requires 1–2 mesh elements per wavelength to ensure computational convergence. To balance accuracy and efficiency, the maximum mesh size in all material domains was uniformly set to 1/1.5 of the shear wavelength (approximately 0.67 wavelengths). This configuration ensures numerical accuracy while effectively controlling computational scale, achieving an optimal balance between precision and efficiency. The final mesh discretization is shown in Figure 3.

## 4. Results and Discussion

### 4.1. Effects of Electrode Width on Piezoelectric Wafer Performance

**Figure 4:**
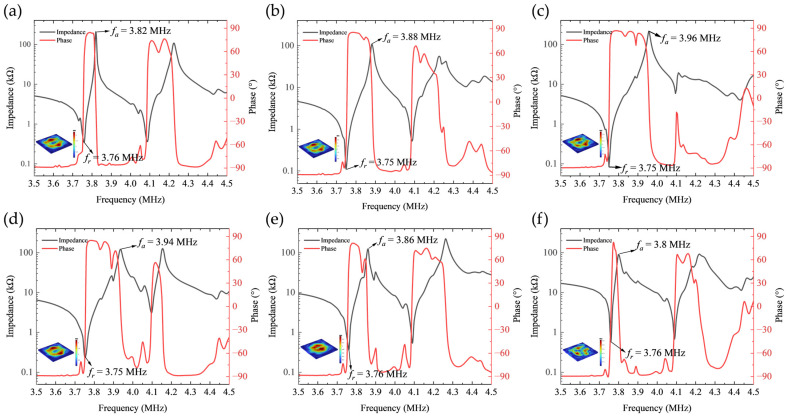
Impedance phase characteristics of the first set of electrode configurations. (**a**) Electrode 1; (**b**) Electrode 2; (**c**) Electrode 3; (**d**) Electrode 4; (**e**) Electrode 5; (**f**) Electrode 6.

The simulated impedance phase curves for the first set of electrode configurations are presented in Figure 4. Analysis reveals that the resonance frequency of the first peak remains relatively stable around 3.75 MHz. As the width of the negative electrode progressively decreases, the bandwidth of this resonance peak exhibits a non-monotonic trend, initially broadening before narrowing, indicating complex evolution of the device’s energy dissipation mechanisms with variations in electrode geometry.

**Figure 5:**
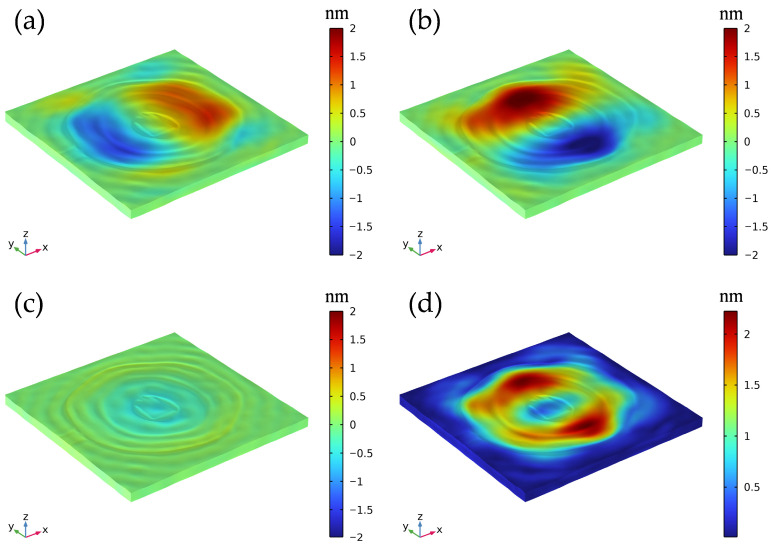
Three-dimensional displacement field distribution of Electrode 4 at the resonance frequency. (**a**) X-direction displacement component $u_{x}$; (**b**) Y-direction displacement component $u_{y}$; (**c**) Z-direction displacement component $u_{z}$; (**d**) Total displacement magnitude.

To gain deeper insight into the physical nature of this primary resonant mode, Figure 5 further illustrates the 3D displacement field distribution of Electrode 4 at its resonance frequency. It can be clearly observed that the displacement component in the thickness direction ($u_{z}$) is significantly smaller than the in-plane displacement components, a characteristic that strongly confirms the dominance of the thickness-shear mode. Furthermore, the displacement component along the Y-axis ($u_{y}$) is notably stronger than that along the X-axis ($u_{x}$), indicating that the Y-axis serves as the dominant vibration direction for this configuration.

Furthermore, a distinct double-peak resonance structure is observable within the 3.5 MHz to 4.5 MHz frequency range. This phenomenon is attributed primarily to the simultaneous excitation of multiple vibration modes and their mutual coupling effects induced by the concentric ring electrode configuration.

As delineated in the theoretical analysis, the non-uniform lateral electric field distribution in the Z-cut lithium niobate wafer effectively excites the thickness-shear mode along the vertical direction through the piezoelectric coefficient $e_{15}$, while concurrently activating the radially extensional mode, governed by the electrode geometry, via the piezoelectric coefficients $e_{31}$ and $e_{33}$.

**Figure 6:**
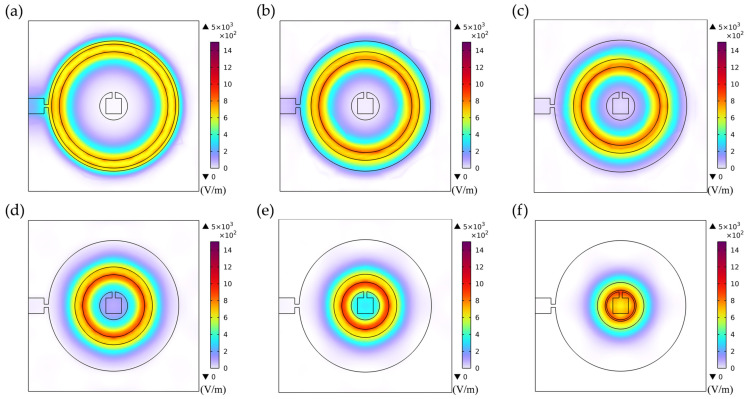
Electric field distribution of the first set of electrode configurations. (**a**) Electrode 1; (**b**) Electrode 2; (**c**) Electrode 3; (**d**) Electrode 4; (**e**) Electrode 5; (**f**) Electrode 6.

Electrode geometric parameters govern the spatial distribution of the lateral electric field, which directly dictates the excitation efficiency and energy localization characteristics of different vibration modes, consequently significantly influencing the resonant response. As shown in Figure 6, with wider electrode rings (e.g., Electrode 1 and Electrode 2), the relatively dispersed electric field distribution not only effectively activates the thickness-shear mode but also strongly excites the radially extensional mode through piezoelectric coefficients $e_{31}$ and $e_{33}$ due to its broader spatial coverage, resulting in a well-separated doublet structure in the frequency spectrum. Under these conditions, the lower degree of electric field energy localization leads to relatively lower energy dissipation for the thickness-shear mode, consequently yielding a narrower bandwidth for its corresponding first resonance peak.

As the electrode rings synchronously contract toward the optimal configuration (e.g., Electrode 3), the electric field distribution becomes markedly localized and concentrated within the central region of the wafer. This enhanced energy confinement effectively improves the electromechanical conversion efficiency of the thickness-shear mode while simultaneously suppressing the excitation strength of the radially extensional mode, thereby altering the modal coupling regime. This transition manifests in the frequency spectrum as a pronounced broadening of the first resonance peak.

When the electrode ring width is further reduced (e.g., Electrode 4 to Electrode 6), the excessively localized electric field distribution, while maintaining high field intensity, exhibits a significantly contracted spatial coverage and substantially reduced effective excitation volume. This change weakens the excitation efficacy for the thickness-shear mode, resulting in a corresponding narrowing of the first resonance peak’s bandwidth.

**Figure 7:**
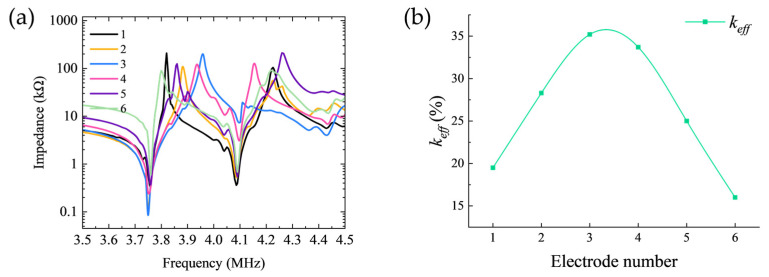
(**a**) Impedance magnitude comparison for configurations with fixed electrode spacing of 0.5 mm. (**b**) Variation of $k_{\mathrm{eff}}$ with electrode geometry for the first set of configurations.

Figure 7a further compares the impedance magnitude curves of the first electrode set. It can be observed that as the negative electrode width decreases, the anti-resonance frequency ($f_{a}$) of the first resonance peak exhibits a non-monotonic trend, initially increasing before decreasing, reaching its maximum value at the configuration corresponding to Electrode 3.

Figure 7b illustrates the variation in the effective electromechanical coupling coefficient ($k_{\mathrm{eff}}$) with electrode geometry for the first set of configurations. Analysis reveals that as the negative electrode width decreases, the $k_{\mathrm{eff}}$ value first increases then decreases, exhibiting a clear optimization window. The maximum $k_{\mathrm{eff}}$ value of 35.2% is achieved with Electrode 3 configuration (*R*_1_ = 0.9 mm, $R_{2}$ = 2.5 mm, $R_{3}\;$= 3 mm, $R_{4}$ = 4.2 mm), which underscores the viability and importance of systematic electrode width optimization for maximizing electromechanical energy conversion efficiency.

### 4.2. Effects of Electrode Spacing on Piezoelectric Wafer Performance

**Figure 8:**
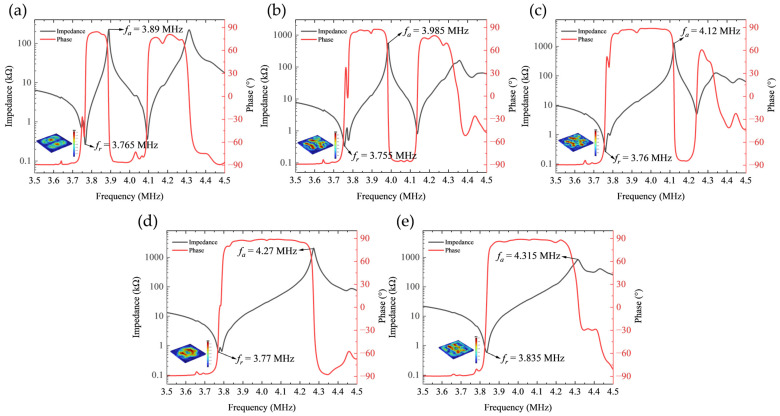
Impedance phase characteristics of the second set of electrode configurations. (**a**) Electrode 7; (**b**) Electrode 8; (**c**) Electrode 9; (**d**) Electrode 10; (**e**) Electrode 11.

The simulated impedance phase responses of the second electrode set are presented in Figure 8. Analysis indicates systematic evolution of impedance characteristics with increasing electrode spacing. The resonance frequency of the first peak remains stable around 3.7 MHz while its bandwidth progressively broadens. Concurrently, the relative magnitude of the second resonance peak appearing within the 3.5–4.5 MHz range decreases significantly. This phenomenon reveals the selective nature of electrode spacing in activating different vibrational modes.

**Figure 9:**
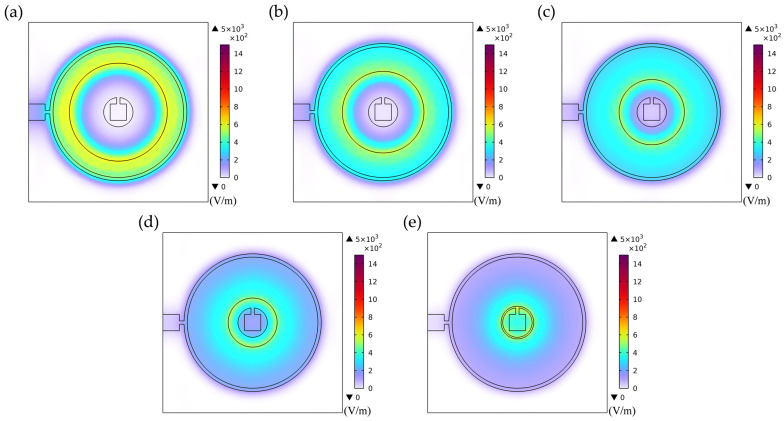
Electric field distribution of the second set of electrode configurations. (**a**) Electrode 7; (**b**) Electrode 8; (**c**) Electrode 9; (**d**) Electrode 10; (**e**) Electrode 11.

At smaller electrode spacings (e.g., Electrode 7), the electric field distribution illustrated in Figure 9a exhibits notably localized characteristics, forming a pronounced lateral electric field confined to the narrow inter-electrode gap region. This highly concentrated field generates strong in-plane stress components along the electrode edges through piezoelectric coefficients $e_{31}$ and $e_{33}$, thereby efficiently exciting the radially extensional mode. Simultaneously, the significant field gradient components effectively couple via $e_{15}$ to activate the thickness-shear mode. When the resonant frequencies of these two modes approach each other under specific parameters, strong modal coupling occurs, manifesting in the impedance spectrum as a distinct double-resonance structure with comparable amplitude and clearly distinguishable peaks.

As the electrode spacing progressively increases (e.g., Electrode 7 to Electrode 10), Figure 9b–e clearly illustrate the evolution of the electric field distribution: the electric field intensity within the inter-electrode gap weakens significantly, the spatial gradient diminishes noticeably, and the field distribution transitions from a highly concentrated state to a more gradual profile. This alteration in field distribution characteristics directly reduces the efficiency of in-plane stress generation via $e_{31}$ and $e_{33}$, substantially suppressing excitation of the radially extensional mode. This suppression manifests in the frequency spectrum as a systematic attenuation of the second resonance peak’s amplitude. Concurrently, although the excitation of the thickness-shear mode is also influenced by the altered electric field distribution, its excitation mechanism via $e_{15}$ exhibits relatively weaker dependence on the field gradient, enabling this mode to maintain comparatively stable excitation efficiency. This selective mode suppression induces a redistribution of the system’s vibrational energy, with increased energy concentration in the thickness-shear mode, thereby achieving a transition from strongly coupled dual-mode vibration to vibration characteristics dominated by the thickness-shear mode.

**Figure 10:**
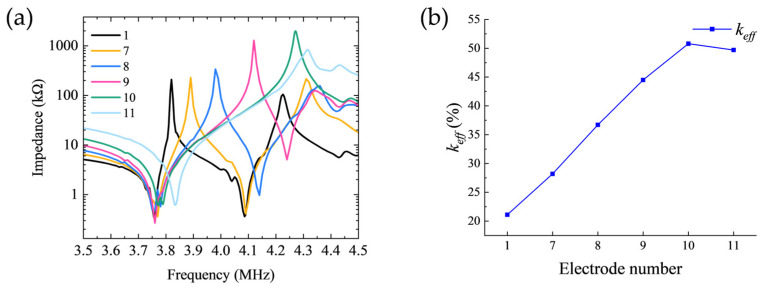
(**a**) Impedance magnitude comparison for configurations with electrode spacing incremented in 0.5 mm steps. (**b**) Variation of $k_{\mathrm{eff}}$ with electrode geometry for the second set of configurations.

Figure 10a compares the impedance curves of the second electrode set. It can be observed that as the electrode spacing increases, the anti-resonance frequency ($f_{a}$) of the first resonance peak exhibits a systematic upward shift. This $f_{a}$ shift is directly linked to the variation in modal coupling strength and significantly influences the electromechanical coupling performance.

Figure 10b illustrates the variation in the effective electromechanical coupling coefficient ($k_{\mathrm{eff}}$) with electrode spacing for the second set of configurations. It can be observed that $k_{\mathrm{eff}}$ exhibits a progressively increasing trend as the electrode spacing expands. This trend indicates that reducing intermodal coupling enhances the electromechanical conversion efficiency for the targeted thickness-shear mode.

Ultimately, a maximum $k_{\mathrm{eff}}$ of 50.8% is attained with Electrode 10 ($R_{1}$ = 0.9 mm, $R_{2}$ = 1.5 mm, $R_{3}$ = 4 mm, $R_{4}$ = 4.2 mm). This result markedly surpasses the optimum value obtained in the first experimental set, conclusively demonstrating that controlling electrode spacing constitutes a more effective strategy for enhancing the electromechanical performance of Z-cut lithium niobate transducers.

### 4.3. Effects of Electrode Width on Ultrasonic Transducer Performance

**Figure 11:**
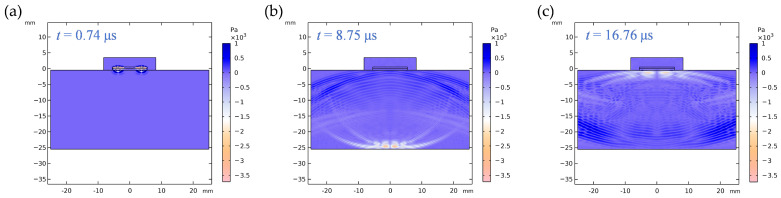
Shear wave (Orange waveform) propagation process in the aluminum block. (**a**) t = 0.74 μs, shear wave enters the top surface of the aluminum block; (**b**) t = 8.75 μs, shear wave reaches the bottom surface of the aluminum block; (**c**) t = 16.76 μs, shear wave returns to the top surface of the aluminum block.

To systematically evaluate the influence of electrode geometry width on the acoustic performance of the transducer, this study conducted acoustic field simulations based on the pulse-echo method using a transducer model integrated with a 25 mm thick aluminum test block. Figure 11 clearly depicts the characteristic transient process of shear wave propagation within the aluminum block: Figure 11a shows the initial incidence of the shear wave (orange waveform) at the top surface of the aluminum block at *t* = 0.74 μs; Figure 11b captures the waveform characteristics as the shear wave propagates to the bottom surface of the aluminum block at t = 8.75 μs; Figure 11c presents the complete scenario at t = 16.76 μs when the shear wave returns to the top surface of the aluminum block.

According to the material parameters listed in Table S4, the shear wave velocity in aluminum is 3120 m/s. Based on acoustic wave propagation theory, the theoretical round-trip propagation time of the shear wave in a sample with thickness d  = 25 mm is determined by the following equation:

(10) $$T=\frac{2d}{v_{s}}$$

where T is the round-trip propagation time, d is the sample thickness, and $v_{s}$ is the shear wave velocity in the material.

Substituting the thickness of the aluminum test block and the shear wave velocity yields a theoretical round-trip time of 16.025 μs, which shows excellent agreement with the simulated round-trip time of approximately 16.02 μs. This close correspondence validates the accuracy and physical soundness of the developed finite element model for simulating complex acoustic wave propagation.

Building upon the validated model, the influence mechanism of electrode width on pulse-echo characteristics was further investigated. In acoustic testing, transducer performance is quantified by analyzing the received echo signal. The signal’s spectrum, obtained via Fast Fourier Transform (FFT), reveals two key parameters: the center frequency ($f_{c}$) and the bandwidth (BW). The center frequency ($f_{c}$) is calculated from the −6 dB drop points using the following equation:

(11) $$f_{c}=\frac{f_{1}+f_{2}}{2}$$

where $f_{1}$ and $f_{2}$ represent the lower and upper frequency limits, respectively, corresponding to the −6 dB points in the normalized echo spectrum.

The −6 dB bandwidth of the transducer is determined by:

(12) $$BW=\frac{f_{2}-f_{1}}{f_{c}}\times 100$$

**Figure 12:**
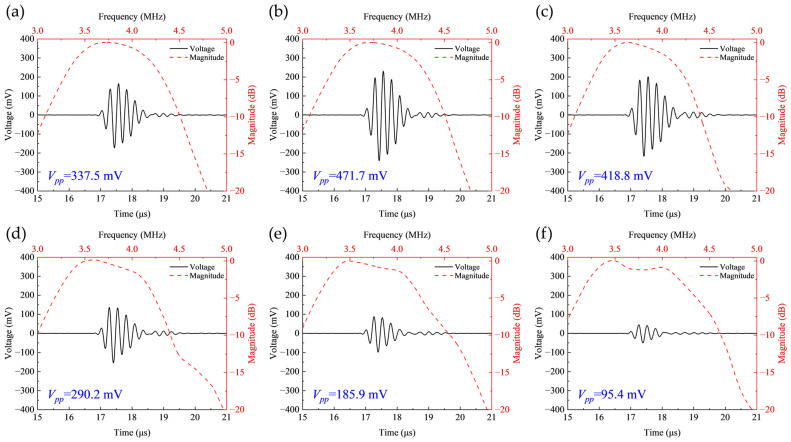
Pulse-echo responses for the first set of electrode configurations. (**a**) Electrode 1; (**b**) Electrode 2; (**c**) Electrode 3; (**d**) Electrode 4; (**e**) Electrode 5; (**f**) Electrode 6.

Based on the quantitative analysis methodology described above, the simulated pulse-echo responses for the first set of electrode configurations are presented in Figure 12.

**Figure 13:**
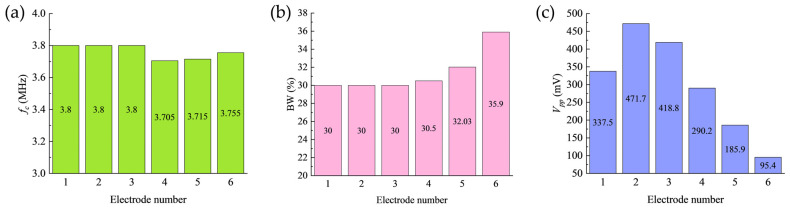
Pulse-echo analysis results for the first set of electrode configurations. (**a**) Center frequency; (**b**) Bandwidth; (**c**) Peak-to-peak echo amplitude.

Systematic characterization was subsequently performed. The results, summarized in Figure 13, demonstrate that with a fixed inter-electrode gap of 0.5 mm, the transducer’s center frequency remains stable at approximately 3.75 MHz across all configurations (Figure 13a), while the −6 dB relative bandwidth also stays around 30% (Figure 13b), indicating good frequency stability.

Notably, the peak-to-peak echo amplitude ($V_{pp}$) of the received shear wave echo exhibits a significant non-monotonic variation as the negative electrode width decreases. The $V_{pp}$ reaches its maximum value of 471.7 mV under the Electrode 2 configuration, while dropping to a minimum of 95.4 mV for the Electrode 6 configuration, as shown in Figure 13c. The maximum $V_{pp}$ exceeds the minimum value by a factor of 4.94.

**Figure 14:**
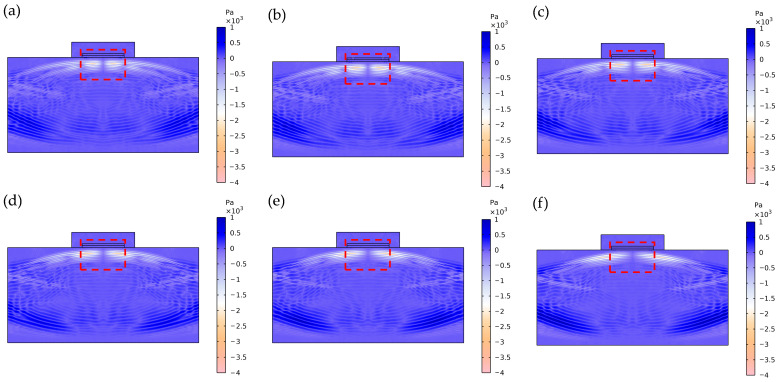
Transient acoustic field distributions of the first set of electrode configurations at t = 16.5 μs. (**a**) Electrode 1; (**b**) Electrode 2; (**c**) Electrode 3; (**d**) Electrode 4; (**e**) Electrode 5; (**f**) Electrode 6.

Concurrently, this study reveals a non-coincident optimization condition in the first electrode set, with maximum $V_{pp}$ and $k_{\mathrm{eff}}$ occurring at Electrode 2 and Electrode 3, respectively. Figure 14 presents the transient acoustic field distributions at t = 16.5 μs for these configurations. Under the fixed 0.5 mm electrode spacing condition, shear wave fields demonstrate progressively broader lateral spreading as electrode width decreases, thereby reducing the acoustic energy effectively received by the piezoelectric layer. Although Electrode 3 achieves the highest $k_{\mathrm{eff}}$, its shear wave field (Figure 14c) exhibits noticeable lateral dispersion, resulting in substantial acoustic energy loss during propagation. In contrast, Electrode 2 not only generates stronger shear wave energy but also maintains superior axial concentration in its acoustic field distribution (Figure 14b). This focused propagation enables more efficient energy recovery after reflection from the aluminum block bottom, consequently yielding the maximum echo amplitude in this configuration set. This phenomenon confirms that the ultimate acoustic output performance is determined by the combined effects of multiple factors including electromechanical conversion efficiency and acoustic radiation characteristics.

### 4.4. Effects of Electrode Spacing on Ultrasonic Transducer Performance

**Figure 15:**
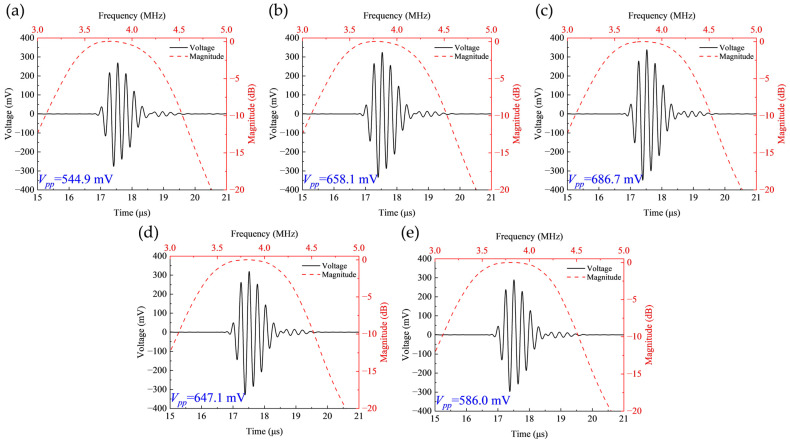
Pulse-echo responses for the second set of electrode configurations. (**a**) Electrode 7; (**b**) Electrode 8; (**c**) Electrode 9; (**d**) Electrode 10; (**e**) Electrode 11.

**Figure 16:**
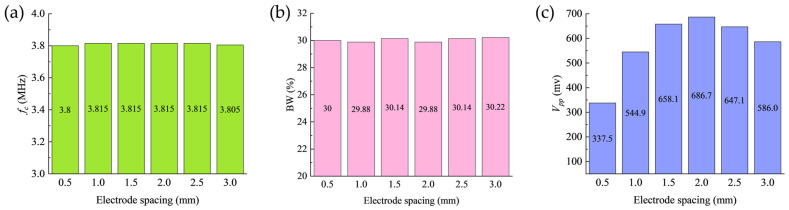
Analysis results of pulse-echo responses for the second set of electrode configurations. (**a**) Center frequency; (**b**) Bandwidth; (**c**) Peak-to-peak echo amplitude.

To investigate the influence of electrode spacing on the acoustic performance of the transducer, systematic pulse-echo simulations were conducted for the second set of electrode configurations. The corresponding echo signals for each configuration are shown in Figure 15, and the extracted performance parameters are summarized in Figure 16.

Analysis results indicate that as the electrode spacing increases from 0.5 to 2.0 mm, the transducer’s center frequency remains stable at approximately 3.8 MHz (Figure 16a), while the −6 dB relative bandwidth maintains around 30% (Figure 16b), exhibiting no significant variation across this parameter range. As shown in Figure 16c, the peak-to-peak echo amplitude ($V_{pp}$) exhibits a distinct correlation with electrode spacing. When the positive-negative electrode spacing increases to 2.0 mm (Electrode 9), $V_{pp}$ reaches its maximum value of 686.7 mV. In contrast, the minimum electrode spacing configuration of 0.5 mm (Electrode 1) yields a $V_{pp}$ of only 337.5 mV, with the maximum amplitude being 2.03 times the minimum value. This observed trend shows close alignment with the increasing tendency of the effective electromechanical coupling coefficient ($k_{\mathrm{eff}}$) versus electrode spacing presented in Figure 10b.

This correlation elucidates the underlying mechanism through which electrode spacing governs acoustic performance: increased spacing modifies the lateral electric field distribution, which suppresses radial vibration mode excitation while simultaneously enhancing the electromechanical coupling of the thickness-shear mode. As shown in Figure 10b, the systematic enlargement of electrode spacing from Electrode 7 to Electrode 10 elevates the $k_{\mathrm{eff}}$ from its initial lower value to a maximum of 50.8%. This enhancement in electromechanical performance directly translates to improved acoustic output capability.

Furthermore, this study observes that the maximum $V_{pp}$ occurs at Electrode 9 while the maximum $k_{\mathrm{eff}}$ appears at Electrode 10, revealing a non-monotonic relationship between acoustic output performance and electromechanical coupling efficiency.

**Figure 17:**
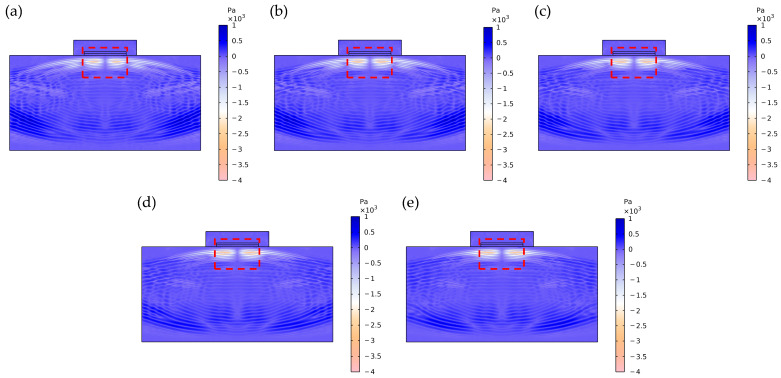
Transient acoustic field distributions of the second set of electrode configurations at t = 16.5 μs. (**a**) Electrode 7; (**b**) Electrode 8; (**c**) Electrode 9; (**d**) Electrode 10; (**e**) Electrode 11.

Figure 17 displays the transient acoustic field distributions of the second electrode set at t = 16.5 μs. As electrode spacing increases, the shear wave field exhibits slight divergence, resulting in somewhat reduced acoustic energy returned to the piezoelectric layer. The configuration corresponding to Electrode 9 demonstrates the strongest echo signal at this moment, as its excited shear wave combines high energy intensity with good axial concentration, enabling most acoustic energy to effectively return to the piezoelectric layer. In contrast, when the spacing further increases to the Electrode 10 configuration, despite achieving maximum electromechanical coupling efficiency, the acoustic field distribution shows certain dispersion with partial shear wave energy laterally dissipated during propagation, consequently yielding slightly lower echo amplitude than Electrode 9. This phenomenon further confirms that the acoustic output performance of transducers depends not only on electromechanical conversion efficiency but is also influenced by acoustic field distribution characteristics.

The results demonstrate that optimizing electrode spacing enables an optimal balance between electromechanical conversion efficiency and acoustic radiation efficiency, providing valuable guidance for designing ultrasonic transducers for different application requirements. Specifically, for applications prioritizing high echo amplitude (e.g., nondestructive testing), electrode configurations that maintain optimal acoustic field concentration should be prioritized to enhance acoustic energy radiation. Conversely, for applications emphasizing bandwidth or filtering performance, electrode designs achieving maximum electromechanical coupling coefficient may be more appropriate.

## 5. Conclusions

This study established a parametric two-dimensional finite element model for Z-cut lithium niobate crystals to systematically investigate the influence of concentric ring electrode geometry on the electromechanical characteristics and acoustic performance of transducers. Through comprehensive frequency-domain impedance analysis and transient pulse-echo simulations, the intrinsic correlations between electrode geometric parameters and transducer performance were revealed. The main conclusions are as follows:

(1) With a fixed electrode spacing of 0.5 mm, the effective electromechanical coupling coefficient ($k_{\mathrm{eff}}$) exhibits a non-monotonic trend, initially increasing and then decreasing as the electrode ring width is reduced. The $k_{\mathrm{eff}}$ reaches its maximum value of 35.2% within this set for the configuration with electrode parameters $R_{1}$ = 0.9 mm, $R_{2}$ = 2.5 mm, $R_{3}$ = 3 mm, and $R_{4}$ = 4.2 mm.
(2) With the electrode spacing progressively increased in 0.5 mm steps, the effective electromechanical coupling coefficient ($k_{\mathrm{eff}}$) exhibits an initial increase followed by a decrease, reaching its maximum value of 50.8% for the configuration with electrode parameters $R_{1}$ = 0.9 mm, $R_{2}$ = 1.5 mm, $R_{3}$ = 4 mm, and $R_{4}$ = 4.2 mm.
(3) With a fixed electrode spacing of 0.5 mm, the peak-to-peak echo amplitude ($V_{pp}$) reaches its maximum value of 471.7 mV for the electrode configuration with parameters $R_{1}$ = 0.9 mm, $R_{2}$ = 3 mm, $R_{3}$ = 3.5 mm, and $R_{4}$ = 4.2 mm, which is 4.94 times that of the minimum value within the same group.
(4) With the electrode spacing progressively increased in 0.5 mm steps, the peak-to-peak echo amplitude attains its maximum value of 686.7 mV for the electrode configuration with parameters *R*_1_ = 0.9 mm, *R*_2_ = 2 mm, *R*_3_ = 4 mm, and *R*_4_ = 4.2 mm, which is 2.03 times that of the minimum value within the same parameter group.

This study confirms that precise control of the concentric electrode ring width and gap spacing effectively optimizes both the electromechanical coupling performance and acoustic wave transmission capability of Z-cut lithium niobate transducers. These findings provide crucial design guidelines for developing high-performance lead-free piezoelectric transducers, offering not only direct application value in industrial contact-based nondestructive testing but also demonstrating broad application prospects in shear-wave-based structural health monitoring. Furthermore, as a foundational simulation-based exploration, this study utilizes the Z-cut orientation as a preliminary model system to reveal the correlation between concentric ring geometry and acoustic energy output. Having established these geometric tuning laws, future work will extend this strategy to other crystal cuts of lithium niobate to explore multimodal excitation capabilities, thereby laying a theoretical foundation for developing high-performance multimodal ultrasonic transducers for complex solid detection or potential hydrophone applications.

## Figures and Tables

**Table 1:** Model boundary conditions.

Boundary	Mechanical Boundary Conditions	Electrical Boundary Conditions
Γ*_damp_T_* (top of damp)	Low reflecting boundary	Zero charge
Γ*_damp_L_*, Γ*_damp_R_* (left and right of damp)	Low reflecting boundary	Zero charge
Γ− (negative electrode)	Free	Ground
Γ+ (positive electrode)	Free	Terminal
Γ*_match_B_* (bottom of match)	Continuity	Zero charge
Γ*_Al_T_* (top of Al)	Free	Zero charge
Γ*_Al_L_*, Γ*_Al_R_* (left and right of Al)	Low reflecting boundary	Zero charge
Γ*_Al_B_* (bottom of Al)	Free	Zero charge

## Data Availability

The original contributions presented in this study are included in the article/Supplementary Materials. Further inquiries can be directed to the corresponding author.

## Supplementary Materials

The following supporting information can be downloaded at: https://www.mdpi.com/article/10.3390/s26020481/s1, Table S1: Geometric parameters for the first set of electrode configurations (fixed inter-electrode gap 0.5 mm); Table S2: Geometric parameters for the second set of electrode configurations (electrode spacing increased in 0.5 mm increments); Table S3: Geometric parameters of the transducer; Table S4: Material properties of the components.
