# Supplementary material for: Mechanisms of Concentric Ring Electrodes in Tuning the Performance of Z-Cut Lithium Niobate Ultrasonic Transducers
Source: Sensors (Basel). 2026 Jan 11;26(2):481. doi: 10.3390/s26020481 (PMC12845614; doi:10.3390/s26020481)
Supplement: Supplementary file 1 [file sensors-26-00481-s001.zip › sensors-3991873-supplementary.pdf]

sensors

MDPI

SUPPLEMENTARY MATERIAL

Mechanisms of Concentric Ring Electrodes in Tuning the Performance of Z-Cut Lithium Niobate Ultrasonic Transducers

Xuesheng OuYang [1], Liang Zhong [1,3], Jun Zhou [1,2,3,*], Guanghua Li [5], Hui Hu [1,2,3], Kai Wang [5], Yizhe Jia [1], Hao Dai [4], Jinlong Mo [1], Kaiyan Huang [1,5] and Jishuo Wang [1]

1 Key Laboratory of Testing Technology for Manufacturing Process MOE, Southwest University of Science and Technology, Mianyang 621010, China
2 School of Mechanical Engineering, Hebei University of Technology, Tianjin 300401, China
3 Sichuan Electronic and Mechanic Vocational College, Mianyang 621023, China
4 School of Information and Control Engineering, Southwest University of Science and Technology, Mianyang 621000, China
5 Henan Key Laboratory of Underwater Intelligent Equipment, the 713th Research Institute of China State Shipbuilding Corporation Limited, Zhengzhou 450000, China
* Correspondence: junez@swust.edu.cn (J.Z.)

Table S1. Geometric parameters for the first set of electrode configurations (fixed inter-electrode gap 0.5 mm).

Electrode Number	R_1(mm)	R_2(mm)	R_3(mm)	R_4(mm)	L(mm)
1	0.9	3.5	4	4.2	1
2	0.9	3	3.5	4.2	1
3	0.9	2.5	3	4.2	1
4	0.9	2	2.5	4.2	1
5	0.9	1.5	2	4.2	1
6	0.9	1	1.5	4.2	1

Table S2. Geometric parameters for the second set of electrode configurations (electrode spacing increased in 0.5 mm increments).

Electrode Number	R_1 (mm)	R_2 (mm)	R_3 (mm)	R_4 (mm)	L (mm)
7	0.9	3	4	4.2	1
8	0.9	2.5	4	4.2	1
9	0.9	2	4	4.2	1
10	0.9	1.5	4	4.2	1
11	0.9	1	4	4.2	1

Table S3. Geometric parameters of the transducer.

Parameters	L_{Al}	H_{Al}	H_{match}	H_{LN}	L_{damp}	H_{damp}	L_1
Dimensions (mm)	50	25	0.5	0.5	16.5	4	11

Table S4. Material properties of the components.

Material	Density (kg/m^3)	Longitudinal Wave Velocity (m/s)	Shear Wave Velocity (m/s)
Tungsten / Epoxy Resin	6580	1500	775
$LiNbO_3$	4700	7360	3980
Al_2O_3/Epoxy Resin	2280	3400	1920
Al	3900	6200	3120
